# Use of unit standard paragraphs and letters in: 1. Australian Familial Cancer Centres 2. One Centre’s experience at Southern Health

**DOI:** 10.1186/1897-4287-10-S2-A52

**Published:** 2012-04-12

**Authors:** C Smyth, C Holding, C Hunt, S O’Connell, E Johnson, N Gelfand, M Harris

**Affiliations:** 1 Southern Health Familial Cancer Centre, Australia

## 

Dictating and checking of letters is a time consuming but essential part of the work of all Familial Cancer Services/Cancer Genetics units.

1. We contacted Familial Cancer Centres/Clinical Genetics units across Australia (17 in total) to ask about the use of unit **standard letters and paragraphs** for Familial Cancer patients in each service. - 14 services provided data. Both Genetic Counsellors and unit typists were contacted where possible:

• 5/14 services had both unit standard letters and paragraphs.

• 3/14 services had neither unit standard letters nor paragraphs.

• 6/14 services had one or the other.

• 5 units had no unit typist although some doctor’s typing was outsourced.

Unit standard letters were used more often than standard paragraphs. These standard letters were regularly used by Genetic Counsellors in the majority of practices across Australia, and infrequently used by unit doctors. However in 3 services, standard letters were used by most staff. The use of standard letters was generally thought to be efficient and led to consistency of information across a department but problems included:

• modifications to the standard template, which typists found reduced time savings;

• lack of personalisation of information;

• poor flow of the letter.

2. Our service has standard paragraphs, but these have not been regularly used. In early 2011 we undertook reformatting (and implementing) standard letters and paragraphs for breast/ovarian patients (80% of our service). Three of our clinic staff (2 genetic counsellors, 1 doctor), who had not previously used standard letters or paragraphs implemented using these reformatted changes with the aim of reducing clinical time checking letters and particularly in our unit, to reduce the typist’s work.

Prior to use of the standard letters/paragraphs, it was estimated the typist took 17 minutes (n=30 letters) to type a patient letter and 10 minutes to type doctor letter (n=19 letters). It took the 3 clinicians an average of 12 minutes (n=36 letters) to check a patient letter and 6 minutes to check a doctor letter (n=28 letters).

Early data of standard paragraphs shows little change in typing time, but some drop in clinician letter checking time. Multiple modifications made to the standard letter template are thought to be the main barrier to a reduction in typing time. We continue to review our use of standard letter templates.

